# Revisiting Aire and tissue-restricted antigens at single-cell resolution

**DOI:** 10.3389/fimmu.2023.1176450

**Published:** 2023-05-03

**Authors:** Minoru Matsumoto, Hideyuki Yoshida, Koichi Tsuneyama, Takeshi Oya, Mitsuru Matsumoto

**Affiliations:** ^1^ Department of Molecular Pathology, Tokushima University Graduate School of Biomedical Sciences, Tokushima, Japan; ^2^ Division of Molecular Immunology, Institute for Enzyme Research, Tokushima University, Tokushima, Japan; ^3^ YCI Laboratory for Immunological Transcriptomics, RIKEN Center for Integrative Medical Science, Yokohama, Japan; ^4^ Department of Pathology and Laboratory Medicine, Tokushima University Graduate School of Biomedical Sciences, Tokushima, Japan

**Keywords:** mTEC, Aire, single-cell analysis, heterogeneity, tissue-restricted antigens

## Abstract

The thymus is a highly specialized organ that plays an indispensable role in the establishment of self-tolerance, a process characterized by the “education” of developing T-cells. To provide competent T-cells tolerant to self-antigens, medullary thymic epithelial cells (mTECs) orchestrate negative selection by ectopically expressing a wide range of genes, including various tissue-restricted antigens (TRAs). Notably, recent advancements in the high-throughput single-cell analysis have revealed remarkable heterogeneity in mTECs, giving us important clues for dissecting the mechanisms underlying TRA expression. We overview how recent single-cell studies have furthered our understanding of mTECs, with a focus on the role of Aire in inducing mTEC heterogeneity to encompass TRAs.

## 1 Introduction

In the thymus, the cortex and the medulla coordinate to elicit proficient T-cells that can effectively eliminate foreign pathogens while simultaneously exhibiting tolerance towards self-components. Basically, cortical thymic epithelial cells (cTECs) orchestrate positive selection, while medullary thymic epithelial cells (mTECs) orchestrate negative selection as well as the induction of regulatory T-cells (Tregs) (1, 2). To effectively screen for an extensive array of self-reactive T-cell clones, mature mTECs are capable of “ectopically” or “promiscuously” expressing nearly 90% of the coding genome, including thousands of tissue-restricted antigens (TRAs) (3–5). Due to their exceptional characteristics, the thymic medulla has been proposed to function as “a mosaic of epithelial self” by mirroring extra-thymic tissues (6, 7).

Since long before, scientists have been aware of the heterogeneity among mTECs as evidenced by histological examinations through electron microscopy. This is exemplified by the identification of multiple subsets such as myoid cells (8), ciliated cells, and secretory cells (9). Besides these morphological variations, further analysis disclosed the nature of promiscuous gene expression (pGE) among mTECs (7), and a seminal study identified autoimmune regulator (Aire) as the key molecule for pGE (10). Notably, individual mTECs do not uniformly express TRA genes in the existence of Aire, but it is estimated that only 1-3% of mTECs would express a particular TRA at any given time (4, 5, 11–13). Consequently, there exist two kinds of heterogeneity among mTECs; (i) inter-populational heterogeneity among mTECs based on the morphology and biological properties and (ii) intra-populational heterogeneity among Aire-expressing mTECs associated with pGE. Although recent transcriptomic analyses have provided various insights into the biology of mTECs, the complexity of mTEC heterogeneity impedes understanding of how mTECs orchestrate the presentation of TRAs as a whole. In this review, we will attempt to unravel this enigma by summarizing the findings in mTECs and Aire brought to us by recent single-cell studies.

## 2 The “two-faced” role of Aire in mTECs

Depending on their surface markers, mTECs have been generally categorized into two distinct subsets; MHC-II^low^CD80^low^ (mTEC^low^) and MHC-II^high^CD80^high^ (mTEC^high^), the latter being considered a mature subset. Along with the high expression of molecules related to antigen presentation, mTEC^high^ is distinguished by the unique expression of Aire in the form of dot morphology in the nucleus (14–16). Initially, the human AIRE gene was positionally cloned as the causative gene for autoimmune polyendocrinopathy-candidiasis-ectodermal dystrophy (APECED), which is characterized by organ-specific autoimmune disease with an autosomal recessive inheritance (17, 18). As for the pathogenesis of the disease, Anderson et al. first demonstrated that TRAs are enriched among the down-regulated genes in mTECs from Aire-knockout (Aire-KO) mice utilizing microarray analysis (10). Based on this result, they suggested that Aire is responsible for the establishment of central tolerance by promoting TRA expression from mTECs. Consistent with this finding, subsequent transcriptomic studies by high-throughput RNA-seq analysis reported that nearly 4,000 genes, including a large number of TRAs, were down-regulated in Aire-KO mTEC^high^ compared with those from wild-type (WT) mTEC^high^ (4, 19).

**Figure 1:**
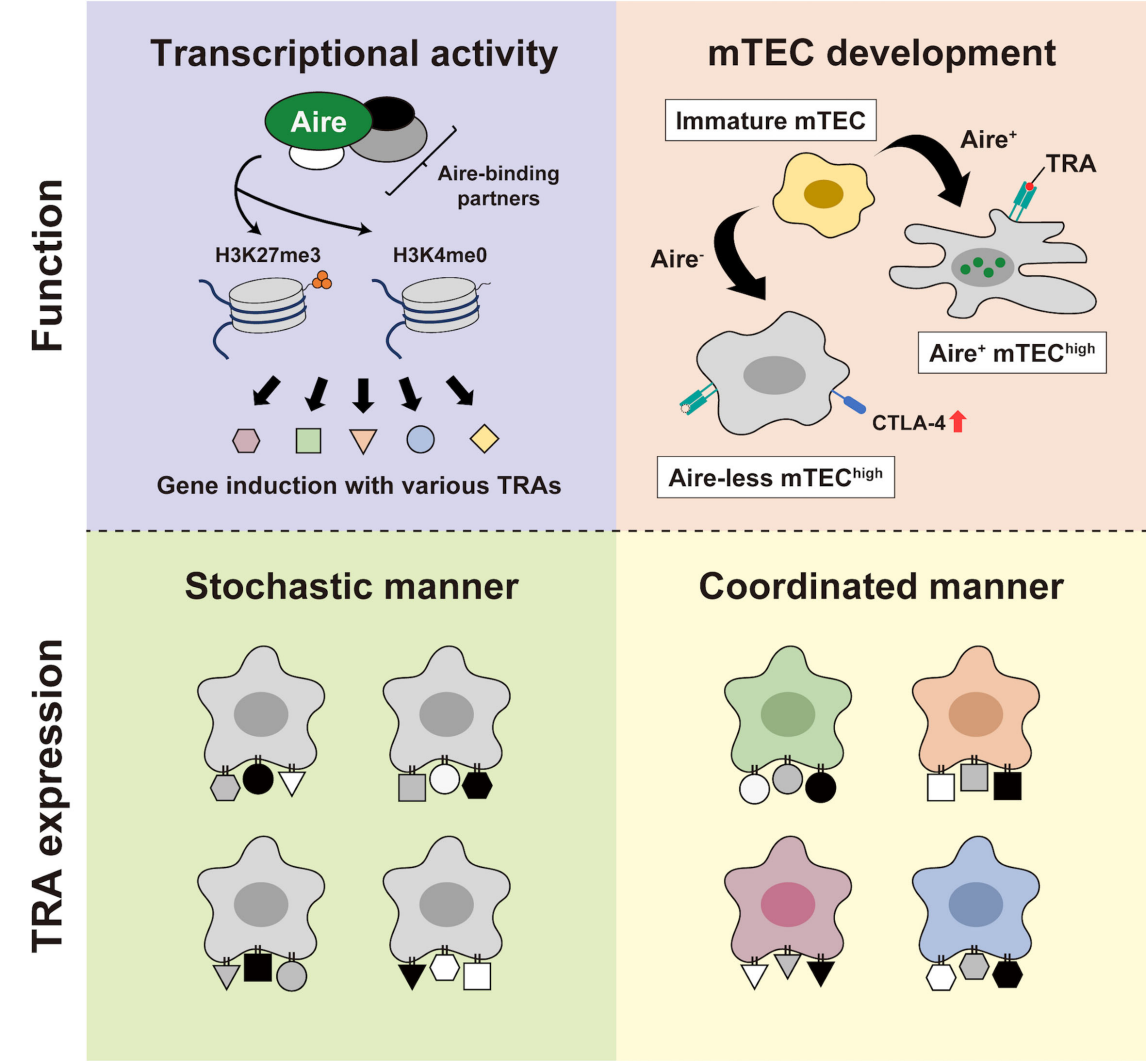
The “two-faced” role of Aire. (*Upper left*) Aire orchestrates its transcriptional function by employing epigenetic mechanisms. Through coordinating with various binding partners, Aire preferentially interacts with repressive chromatin marks, such as H3K4me0 and H3K27me3, thereby derepressing gene expression. (*Upper right*) Aire’s participation in the mTEC development. In the presence of Aire, mTEC^high^ attains a fully developed capability with abundant TRA expression. Conversely, Aire-deficiency leads to the altered development of mTEC^high^ with ectopic CTLA-4 expression and a defect in TRA expression. (*Lower left* and *lower right*) Depicting two potential paradigms for TRA expression; a stochastic manner without any coherent biological relevance (*left*) and a coordinated manner based on the biological properties of particular types of mTEC (*right*).

Then, how does a single Aire gene elicit such a dynamic alteration in the transcriptome? The current prevailing view is that Aire operates as a transcription factor to promote a broad array of transcriptional targets, not by recognizing particular DNA sequences but by involving epigenetic mechanisms. It has been suggested that Aire preferentially engages in the interaction with repressive chromatin states, such as H3K4me0 and H3K27me3 (4, 20–23), in cooperation with various partner molecules (24–29) (**Figure 1**, *upper left*). Upon being recruited to such a chromatin state, Aire is considered to promote the transcription of TRA genes by releasing stalled polymerase II from their promoters (30, 31). Furthermore, a recent study using ChIP-seq demonstrated that Aire-containing complexes are preferentially located on super-enhancers, chromatin stretches hosting high densities of general and cell-type-specific transcription factors, to induce TRAs (32). However, the function of Aire turned out to extend beyond simply serving as a transcriptional regulator of TRA genes, suggesting that it plays a pivotal role in the development/differentiation of mTECs (**Figure 1**, *upper right*). This notion was first derived from observing morphological alteration in the medullary components in Aire-KO mice (33, 34). Aire-KO mice were reported to exhibit increased numbers of mTECs with a globular cell shape (35, 36) and a near absence of Hassall’s corpuscle-like structures (36, 37), along with increased percentages of mTEC^high^ (38–41). Recently, we and another group have also reported that mTEC^high^ from Aire-KO mice ectopically expresses CTLA-4 at a high level (42, 43). Thus, the Aire-KO mTEC^high^ is not only defective in TRA expressions but also impaired in their normal development, which would be required for tolerogenic function.

Besides this “two-faced” mode regarding the role of Aire (i.e., transcriptional activity and mTEC development), there also exist two potential paradigms in debate for the way in which mTECs present TRAs to T-cells in the thymus. The first is a pattern in which individual mTECs express various TRAs in a completely “stochastic” manner, lacking any coherent functional/developmental relevance (**Figure 1**, *lower left*). The second is a pattern in which TRAs are expressed by particular mTECs in a “coordinated” manner based on their biological properties (**Figure 1**, *lower right*). Comparing these two paradigms with the two-faced function of Aire, the stochastic model seems to be more closely linked with pGE due to Aire’s genuine transcriptional activity, whereas the coordinated model appears to pertain to the engagement of Aire in mTEC development. So far, the role of Aire has been basically discussed in a dichotomous manner; “(genuine) transcriptional activity” versus “mTEC development” and “stochastic gene induction” versus “coordinated gene induction.” However, upon considering the findings of prior studies, it seems reasonable to assume that Aire fulfills both of these capacities. We discuss below the multifaceted nature of Aire as revealed by the single-cell analyses.

## 3 Heterogeneity of mTECs revealed by single-cell analysis

Like mTEC^high^, the mTEC^low^ fraction also appears to consist of non-homogenous subsets. Using ontogenetic analysis and reaggregate thymic organ culture (RTOC) system, Gray et al. demonstrated that the mTEC^low^ compartment comprises progenitors developing into mature mTEC^high^ (38). For this developmental process of mTECs, they require multiple TNF receptor superfamily signals, including RANK, CD40 and lymphotoxin-β receptor (44–51). Furthermore, fate-mapping and lineage-tracing studies revealed that the mTEC^low^ fraction also holds mTECs in a terminally differentiated stage, termed “post-Aire” mTECs, that have progressively lost their expression of MHC-II, CD80, and Aire (52, 53). These studies suggested that the classification into mTEC^high^ and mTEC^low^ is insufficient to capture the biology of mTECs.

In addition to these notions, recent advancements in transcriptomic analysis via single-cell RNA-seq (scRNA-seq) have extended our understanding of mTEC heterogeneity. We have summarized previous reports utilizing single-cell analysis, specifically those pertaining to TECs, with their significance in **Table 1**. scRNA-seq has now become a widely employed technique for identifying cell types or cell states in the field of immunology (70). Indeed, scRNA-seq studies have uncovered multiple mTEC subpopulations based on their transcriptome, exemplified by the identification of a novel mTEC subset reminiscent of tuft cells in the small intestine (56, 57). Based on the transcriptome similarity computed by scRNA-seq, Bornstein et al. divided mTECs into four subgroups referring to the previously established mTEC subsets; mTEC I (CCL21^+^ mTEC^low^), mTEC II (mature mTEC^high^), mTEC III (post-Aire mTECs), and mTEC IV (thymic tuft cells) (56). Other single-cell studies also reproduced the identification of thymic tuft cells, not only in mice (59, 61, 62, 64–69) but also in the human thymic stroma (60, 63). Notably, the development of thymic tuft cells is dependent on the transcription factor *Pou2f3*, which serves as a master regulator of intestinal tuft cells (56, 57). The identification of thymic tuft cells reminded us of the model that the thymus comprises “a mosaic of epithelial self.” Besides scRNA-seq, single-cell ATAC-seq (scATAC-seq) has been recently developed as a means to infer the *cis*- and *trans*- gene regulatory mechanisms in single cells in a massively parallel way (71).

**Table 1:** Reports of single-cell approach to TECs including both scRNA-seq and scATAC-seq analyses.

Authors	Organism	Platform	Subject	Significance
Sansom et al., 2014 (4)	Mouse	Fluidigm C1 Smart-seq	MHC-II^high^ mTECs	Identifying Aire-induced genes and uncovering their sparse yet strong expressions within individual mTECs
Brennecke et al., 2015 (5)	Mouse	Smart-Seq2	MHC-II^high^ mTECs, Ceacam1^+^ mTECs, Klk5^+^ mTECs, Tspan8^+^ mTECs	Demonstrating coordinated patterns in TRA expression with their probable association with chromatin accessibility
Meredith et al., 2015 (13)	Mouse	CEL-Seq	MHC-II^high^ mTECs	Detecting diminutive co-expression clusters in Aire’s target genes, characterizing their fashion as “ordered stochasticity”
Miragaia et al., 2018 (54)	Mouse	Fluidigm C1 Smart-seq2	Total mTECs	Analyzing the acquisition process of TRA expression in correlation with the developmental stages of mTECs
Kernfeld et al., 2018 (55)	Mouse	Drop-seq	Total TECs	Uncovering the heterogeneity and transcriptomic dynamics of TECs during thymus organogenesis in embryos
Bornstein et al., 2018 (56)	Mouse	MARS-seq	Total mTECs	The identification of thymic tuft cells as a novel subset of mTECs, with evidence of their dependency on *Pou2f3*
Miller et al., 2018 (57)	Mouse	Smart-seq2	Thymic tuft cells	
Zeng et al., 2019 (58)	Human	Chromium STRT-seq	Total TECs	Characterizing TEC populations during embryonic and fetal stages with the anticipation of intercellular interactions
Dhalla et al., 2020 (59)	Mouse	Chromium	Total mTECs, TSPAN8^+^ mTECs, GP2^+^ mTECs	Identifying ordered co-expression patterns among TRA genes with uncertain biological significance in mTECs
Park et al., 2020 (60)	Human	Chromium	Total TECs	Construction of the comprehensive cell atlas of the thymus, comprising TECs, across the prenatal and postnatal stages
Baran-Gale et al., 2020 (61)	Mouse	Smart-seq2	Total TECs	Unveiling the effect of aging on the TEC composition associated with the disruption of progenitor differentiation
Wells et al., 2020 (62)	Mouse	Chromium	Total TECs	Proposing a branching development into Aire- and Ccl21a-expressing mTECs from transit-amplifying TECs
Bautista et al., 2021 (63)	Human	Chromium	Total TECs	Characterizing thymic stromal cells across different stages of life and detecting unique subsets among mTECs
Nishijima et al., 2022 (64)	Mouse	Chromium	Total TECs	Revealing composition of mTECs dramatically alters in Aire-deficient mice, leading to the impairment of TRA expression
Gao et al., 2022 (65)	Mouse	Chromium	Total TECs	Profiling the temporal dynamics of TEC development and transcriptomics throughout embryonic to adult stages
Miyao et al., 2022 (66)	Mouse	Chromium RamDA-seq	Total TECs (RNA & ATAC) RTOC (RNA)	Demonstrating that transit-amplifying Aire^+^ mTECs serve as precursors of functionally mature Aire^+^ mTECs
Nusser et al., 2022 (67)	Mouse	CEL-Seq2	Total TECs	Identifying two distinct types of principal TEC progenitors by utilizing a CRISPR-Cas9-based barcoding system
Michelson et al., 2022 (68)	Mouse	Chromium	MHC-II^high^ mTECs (ATAC), MHC-II^low^Pdpn^-^CD104^-^ mTECs (RNA)	Suggesting mTECs co-opt lineage-defining transcription factors to mirror extra-thymic cell types for TRA expression
Liang et al., 2022 (69)	Mouse	Chromium	Total TECs	Demonstrating TEC-specific deletion of *Furin* leads to a decrease in the number of mTECs, including thymic tuft cells

## 4 Altered composition of mTECs due to Aire deficiency

Michelson et al. have recently extended the idea described above by demonstrating that mTECs utilize lineage-defining transcription factors to mirror the extra-thymic cell types (68). They conducted a large set of single-cell experiments, including scATAC-seq and scRNA-seq, to investigate the mechanisms of TRA expressions in mTECs. They categorized mTECs into various subpopulations based on their resemblance to particular extra-thymic tissues, such as keratinocytes, tuft cells, microfold cells, neuroendocrine/secretory cells, ciliated cells, and myocytes. They called these populations “mimetic cells” and demonstrated that the majority of them are in the “post-Aire” stage. Similar to thymic tuft cells, they discovered that microfold mTECs are nearly eliminated in mice lacking *Sox8* or *Spib*, transcription factors that characterize the corresponding subset (i.e., microfold cells) (68). Thus, their findings support the concept of coordinated machinery underlying the expression of TRAs (**Figure 1**, *lower right*). Several studies have also reported matched mTEC clusters for mimetic cells, albeit with slight variations in terminology (**Table 2**). Importantly, Aire-KO mice showed the reduction of certain mimetic cell clusters to varying degrees, suggesting that Aire is partially and variably required for the development of these subpopulations (68).

**Table 2:** mTEC subpopulations mirroring extra-thymic cell types identified by single-cell analysis.

Subset	Organism	Gene signature	Other description	Reference
Post-Aire mTEC	Mouse	Spink5, Ivl, Krt10, Krt80, Pigr, Ly6d, Cnfn, Flg	mTEC-III, Keratinocyte mTEC, Corneocyte-like mTEC, Spink5^+^ cell	(56, 59–69)
	Human	KRT1, KRT10, IVL		
Thymic tuft cell	Mouse	Pou2f3, Dclk1, Avil, Lrmp, Trpm5, Il25, Gng13, L1cam, Sox9, Chat	mTEC-IV, Tuft-like mTEC	(56, 59–69)
	Human	POU2F3, DCLK1, GNAT3, GNB3, PLCB2, OVOL3		
Microfold mTEC	Mouse	Sox8, Spib, Gp2, Ccl6, Ccl9, Ccl20, Ccr5, Tnfaip2, Tnfrsf11b	Gp2-preffered mTEC, Gp2^+^ TEC, Ccl6^+^ cell	(59, 65, 66, 68)
Neuroendocrine/ Secretory cell	Mouse	Snap25, Stxbp5l, Car8, Cd177	Structural TEC, TEC (neuro)	(60, 61, 63, 66, 68)
	Human	NEUROD1, NEUROG1, CHGA, BEX1		
Ciliated cell	Mouse	Dynlrb2, Dnah12, Spag16, Wdr34, Bbs7, Tppp3, Fam183b	Cilia-TEC	(59, 63, 66, 68)
	Human	ATOH1, GFI1, LHX3, FOXJ1		
Myoid cell	Mouse	Myog, Myl1, Actc1	Muscle mTEC, TEC (myo)	(60, 63, 68)
	Human	MYOD1, MYOG, DES		
Lung mTEC	Mouse	Aqp4, Aqp5, Muc5b, Slc12a2, Bpifa1	Bpifa1^+^ cell	(65, 68)
Ionocyte	Mouse	Slc12a2, Atp6v1b1		(63, 68)
	Human	CFTR, FOXI1, ASCL3, CLCNKB		
Enterocyte/ Hepatocyte mTEC	Mouse	Reg3g, Saa1, Saa3, Aldob		(68)
Myelin^+^ cell	Human	SOX10, MPZ, MBP, S100A1		(63)

Our previous scRNA-seq study further focused on transcriptome changes in the primary Aire-expressing mTECs caused by the lack of Aire (64). Interestingly, a comprehensive analysis of WT and Aire-KO mice revealed a significant alteration in the composition of mTECs, as also documented in other reports (56, 68). The most striking change was the presence of clusters unique to Aire-KO mTECs that emerged instead of Aire-expressing mTEC^high^ in WT mice; “Aire-less” mTEC^high^, hereafter. These clusters were reminiscent of morphologically abnormal mTECs observed in the medulla in Aire-KO mice (35, 36), and they exhibited high expression of *Ctla4* (42). Besides the defect of TRA genes in Aire-less mTEC^high^ compared with Aire-expressing mTEC^high^, a comparison of these two groups identified a signature of “keratinocyte differentiation,” suggesting the impairment of normal developmental process in Aire-less mTEC^high^ (64). Thus, recent studies utilizing single-cell techniques and Aire-KO mice have indicated that Aire deficiency leads to the impaired development of mTECs at the Aire-expressing stage itself, as well as subpopulations mimicking the extra-thymic tissues to a variable extent. However, the exact mechanisms through which Aire promotes the development of various mTEC subpopulations remain elusive.

## 5 Aire-induced genes revisited by single-cell resolution

As aforementioned, Sansom et al. reported the down-regulation of nearly 4,000 genes in Aire-KO mTEC^high^ in comparison to the wild-type counterpart (4). They classified these genes as “Aire-induced genes” and further defined a group of genes whose expression is entirely dependent on Aire as “Aire-dependent genes.” Although these gene lists have been extensively employed in subsequent studies with the assumption that Aire directly regulates their transcription within Aire-expressing mTECs, the following single-cell studies have provided an alternative interpretation.

Regarding the mTECs in WT mice, a previous single-cell analysis demonstrated that MHC-II^high^ fraction comprises Aire-expressing mTECs and various mTEC subsets mirroring extra-thymic cell types (i.e., mimetic cells) (68). Furthermore, it has been reported that Aire-expressing mTECs in active cell-cycle, known as transit-amplifying TECs (TAC-TECs), also exist within the mTEC^high^ population (62, 66). While these cells were suggested to be the precursors of quiescent and functionally mature Aire-expressing mTEC^high^, they were incapable of expressing sufficient levels of TRA genes together with Aire-induced genes (66).

Notably, compositional changes are taking place in the mTEC^high^ fraction from Aire-KO mice. First, Aire-KO mTECs harbor “Aire-less” mTEC^high^, which shows the defect of TRA expressions (64), altered globular morphology (35, 36) along with the enlarged cell numbers (38–40, 64), and abnormal *Ctla4* expression (42, 43). Second, several mimetic cell clusters were reported to decrease in various degrees (e.g., ciliated mTECs, neuroendocrine/secretory mTECs, and microfold mTECs) (68). In contrast to functionally mature Aire-expressing mTEC^high^, the effect of Aire deficiency in the TAC-TEC cluster seems to be minimal, as we observed no significant changes in their distribution and cell numbers in Aire-KO mice (64).

**Figure 2:**
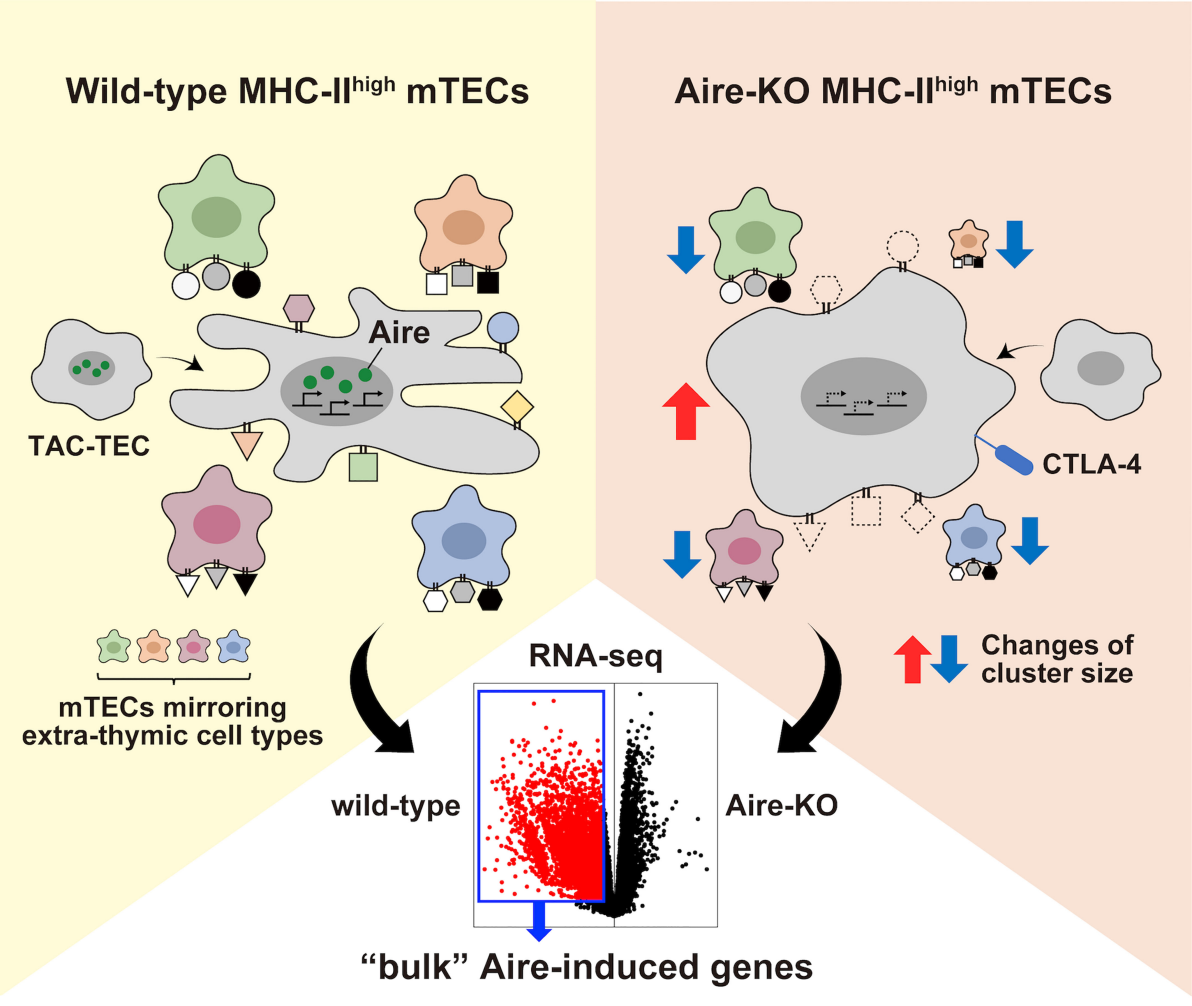
The landscape of the bulk Aire-induced genes. Illustrating the compositional changes between wild-type MHC-II^high^ mTECs and Aire-KO MHC-II^high^ mTECs. Besides the defect in the transcriptional activity in primary Aire-expressing mTEC^high^, the altered composition of various mTEC subpopulations in mTEC^high^ contributes to the difference in the transcriptome detected by the bulk RNA-seq.

When the transcriptomes of mTEC^high^ from WT and Aire-KO mice are compared by bulk RNA-seq, differentially upregulated genes in WT mTEC^high^ would be detected as Aire-induced genes (**Figure 2**). Therefore, “bulk” Aire-induced genes do not constitute a single group of genes brought by the genuine transcriptional activity of Aire. We suggest that the “bulk” Aire-induced genes consist of two contexts in which Aire promotes the genes via distinct regulatory mechanisms; (i) genes induced by Aire’s genuine transcriptional activity and (ii) genes preferentially expressed in mimetic cells. In other words, the higher a mimetic cell cluster’s dependence on Aire, the more genes characteristic of the corresponding cluster will be detected as Aire-induced genes.

## 6 Stochastic “and” coordinated manner in TRAs?

Initially, single-cell analyses with single-cell PCR were applied to MHC-II^high^ mTECs, focusing on certain TRAs to dissect the mechanism underlying TRA expressions (11, 12, 72). Although these studies emphasized the stochasticity of pGE in individual mTECs, later scRNA-seq experiments utilizing a few hundred MHC-II^high^ mTECs concluded that Aire-induced genes fall into some sort of coordinated patterns besides its stochasticity (4, 5, 13). Furthermore, a recent large-scale scRNA-seq analysis (total of 6,894 mTECs) also noted an ordered co-expression pattern in TRAs, confirmed by focusing on particular Aire-induced TRAs (i.e., Tspan8 and Gp2), while the biological significance of co-expression remained inexplicable (59). It should be noted that these arguments are predicated upon the assumption that TRA induction is primarily brought by a single mechanism. However, as discussed above, it seems reasonable to posit that Aire induces TRA expression through a combination of (i) a stochastic pattern resulting from the genuine transcriptional activity of Aire and (ii) a coordinated pattern established by mTECs mirroring extra-thymic cell types (i.e., mimetic cells). Given this two-faced role of Aire, analyses utilizing total mTEC^high^ or focusing on specific TRAs highly expressed within a particular mimetic cell cluster may not be sufficient. Instead, it might be essential to classify mTECs accurately into subclusters in advance and then investigate the significance of Aire-induced genes or TRA genes within individual clusters. In this regard, single-cell analysis, especially the droplet-based approach, is an ideal tool to dissect inter-populational heterogeneity underlying mTECs.

However, since the sequencing depth in each cell is relatively low in the droplet-based method, it may not be sufficient to detect pGE in mTECs due to its sparse nature. To capture the dynamics of the transcriptome in single mTECs, another plate-based approach, such as Smart-seq (73) and RamDA-seq (74), would be better than the droplet-based method as it offers much higher sensitivity and near-complete full-length transcript coverage. Furthermore, the method employed for cell isolation would significantly affect the outcome of downstream analysis. Indeed, a previous study utilizing scRNA-seq for FACS-sorted MHC-II^low^Pdpn^−^CD104^–^-mTEC^low^ fraction (68) detected various mimetic cells at a higher resolution compared with other studies utilizing the total TEC population (62, 64–66). On the other hand, the protocol of TEC isolation itself would also have a significant impact on the result of single-cell analysis (75, 76). Given that a previous imaging study has reported ~1.1x10^6^ Aire^+^ mTECs in 5 weeks old mice (77), it is possible that there still exists unknown TEC populations. The integration of these techniques and methodologies will lead to a better understanding of inter-populational heterogeneity in TECs and intra-populational heterogeneity in Aire-expressing mTECs.

## 7 Shared and distinct features between mouse and human mTECs

Although previous scRNA-seq studies have demonstrated the similarities between mouse and human mTECs, certain differences also exist between them. Representative mTEC clusters corresponding to CCL21^+^ mTEC^low^, AIRE-expressing mTEC^high^ and post-AIRE mTECs were identified among human TECs (60, 63). Several mimetic cell clusters, such as thymic tuft cells, myoid cells and ciliated cells have also been detected both in mouse and human thymic stroma (**Table 1**). Nevertheless, the composition of mimetic cell clusters varies between mice and humans. For example, the cluster of thymic tuft cells was relatively small and indistinct in human mTECs compared with mouse mTECs (60, 63). In contrast, Bautista et al. reported the presence of myelin-expressing mTECs in humans that have not been reported in the mouse thymi (63). Furthermore, the TAC-TEC cluster has not been captured in the previous studies related to human TECs (60, 63). However, we also need to consider the dynamics of TEC composition associated with aging and other stress (61) when comparing mouse and human TECs. Since the single-cell studies on human TECs are currently limited, future studies employing larger sample sizes, higher sequencing depth, and more sophisticated analytical tools will be crucial for elucidating the full spectrum of cellular heterogeneity in human TECs.

## 8 Single-cell analysis beyond mTECs

### 8.1 Aire expression from antigen-presenting cells in the periphery

Other than mTECs, Gardner et al. characterized Aire-expressing hematopoietic population in peripheral lymphoid tissues as extrathymic Aire-expressing cells (eTACs), showing the characteristics of MHC-II^high^ antigen-presenting cells (78, 79). A recent study utilizing single-cell multi-omics (scRNA-seq + scATAC-seq) further divided eTACs into two distinct types; migratory dendritic cell (DC)-type and RORγt^+^ group 3 innate lymphoid cell (ILC3)-type, as they termed the latter Janus cells (JCs) (80). The result was also corroborated by three back-to-back studies that identified Aire-expressing lineage among RORγt^+^ cells by scRNA-seq (81–84). Notably, Dobeš et al. demonstrated that Aire deficiency in ILC3-type eTACs (i.e., JCs) leads to the impaired generation of *Candida*-specific T-cell response, providing new insight into the mechanism underlying mucocutaneous candidiasis of APECED patients and extra-thymic function of Aire (85).

Given that the transcriptional impact of Aire varies with cell type (86), it is questionable whether the presence of Aire elicits the expression of TRAs in hematopoietic cells similar to its effect in mTECs. Wang et al. suggested the similarity in the transcriptome between JCs and mTECs by calculating the cosine similarity score and demonstrated the enrichment of TRAs in JCs, while the effect of Aire deficiency was not mentioned (80). Another study revealed that Aire deficiency impacted the transcriptome under the heat-killed *C. albicans* (HKCA)-stimulated condition in JCs, resulting in the impaired induction of genes encoding cytokines (*Il6*, *Il18* and *Bmp2*), *C. albicans*-sensing receptors (*Clec7a*), cell adhesion molecules (*Vcam1* and *Cadm1*), costimulatory molecules (*Cd86*) and enzymes involved in proinflammatory response (*Ptgs2*) (85). However, the transcriptome changes were not as pronounced as those observed in Aire-KO mTECs. In contrast, we noted no considerable effect on the transcriptome of DC-type eTACs as a result of the lack of Aire (87). Despite these equivocal findings regarding the transcriptional activity of Aire in antigen-presenting cells, a comprehensive study of Aire in distinct cell types and its effect may improve our understanding in the biology of Aire.

### 8.2 AIRE expression in thymic epithelial tumors

Recently, two groups independently reported the scRNA-seq analysis of thymic epithelial tumors (TETs) (88, 89). TETs are classified into six types of thymomas (Type A, AB, B1-B3 thymomas, and micronodular thymoma with lymphoid stroma) and thymic carcinoma according to the current WHO classification (90). While there is no doubt that TETs originate from TECs, it is still elusive whether each histological type originates from cTECs or mTECs. A previous immunohistochemical study reported that most thymomas showed characteristics of bi-lineage differentiation toward mTECs and cTECs, suggesting that they derive from a common progenitor cell (91). Indeed, the scRNA-seq data showed that each TET contained varying proportions of neoplastic TECs resembling normal cTECs and mTECs within the tumor, containing a small number of AIRE-expressing cells (88, 89). In great contrast, our study combining immunohistochemical analysis for AIRE with published scRNA-seq data demonstrated that most thymic carcinomas express AIRE protein and harbor the molecular characteristics of several subpopulations in mTECs, but not cTECs, suggesting their cell of origin as mTECs (92). A recent report also demonstrated that a tuft cell-like signature was prevalent in thymic carcinomas (93).

Patients with thymomas often develop autoimmunity, most typically myasthenia gravis (MG), whereas thymoma is recorded in only 10-15% of all patients with MG (94–96). Besides thymomas, thymic hyperplasia (thymic follicular hyperplasia), which forms abundant germinal centers in the medulla, can also cause MG. Of note, autoimmunity is significantly rare in patients with thymic carcinomas (94, 95). Although the role of AIRE and mTECs in the pathogenesis of MG is still elusive, the absence or the insufficiency of AIRE expression from thymoma tissues has been discussed with the development of autoimmunity in thymoma patients in several studies (97–100). However, our immunohistochemical approach revealed that most type B thymomas, in which paraneoplastic autoimmunity is most frequent, harbored focal but intense AIRE staining in the area showing differentiation to mTECs (92). Furthermore, we did not see a correlation between the incidence of MG and the expression of AIRE protein in our TET cohort, raising a possibility that some other factors than AIRE might be responsible for the development of MG in thymomas. In this regard, it is quite important to disclose whether AIRE is also functional in the neoplastic milieu. While the information on AIRE-dependent genes in humans is currently unavailable, single-cell data accumulated so far from human TECs may help to identify the putative AIRE’s targets. Furthermore, spatial transcriptomics may provide novel insights into the biology of TETs by capturing their abnormal architecture and altered gene expressions simultaneously.

## 9 Conclusions and perspectives

Although recent advances in the single-cell analysis have expanded our understanding of mTECs, it is an issue of great importance for further study to reveal how a single Aire gene participates in such a multilayered task outlined above and how it functions even in the extra-thymic cellular context. It should also be noted that scRNA-seq analysis of embryofetal and neonatal mice draws dramatically different distributions of TECs from adult samples (56, 65). Considering the distinct profiles of Tregs generated in early life (101) and the importance of Aire expression during the perinatal period (102), it would be worthwhile to focus on Aire’s actions during early life to see any possible difference from those seen in adults at a single-cell resolution. It is also crucial to clarify how the TRAs from Aire-expressing mTECs and mimetic clusters share roles for inducing immune tolerance. Particularly, the benefit of stochastic TRA expression from a small population in Aire-expressing mTECs is of great interest. We hope that our review will contribute to a better understanding of mTEC biology and illuminate a new perspective on the Aire.

## Author contributions

MinM wrote the manuscript draft in consultation with KT and TO for improvement. HY and MitM supervised the writing of the manuscript. All authors contributed to the article and approved the submitted version.
